# Breast Reconstruction

**Published:** 2013-01-28

**Authors:** Jennifer E. Kim, Justin M. Broyles, Sachin M. Shridharani, Justin M. Sacks

**Affiliations:** Department of Plastic and Reconstructive Surgery, The Johns Hopkins University School of Medicine, Baltimore, Md

**Figure:**
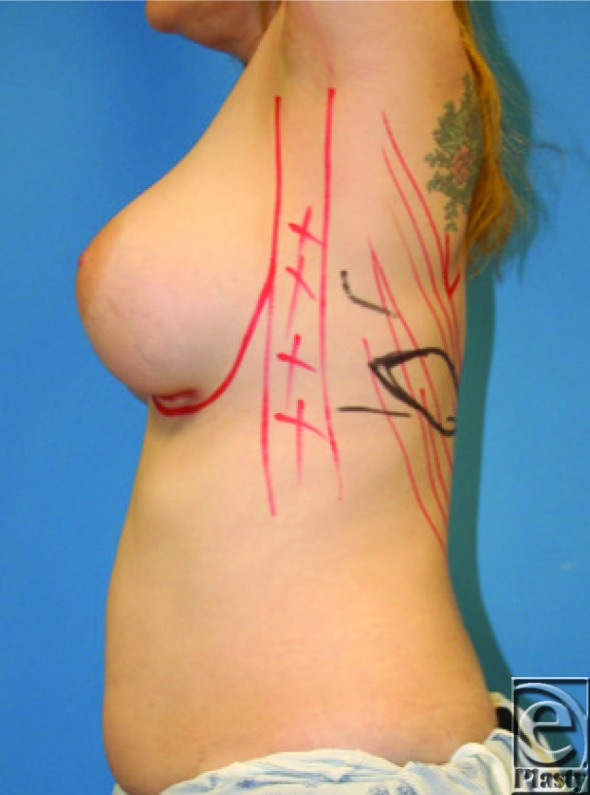


## DESCRIPTION

A 43-year-old woman presents for a preoperative visit before undergoing a bilateral mastectomy. After consultation, she desires autologous tissue reconstruction with maintenance of her current breast size. On examination, she has a 36C bust and has minimal abdominal subcutaneous tissue.

## QUESTIONS

1. **What are the advantages and disadvantages of the latissimus dorsi (LD) flap?**
2. **Who is a candidate for LD flap reconstruction? What are the contraindications?**
3. **Describe the standard operative protocol.**
  **When is an implant used?**
  **What is the effect of the operation on the back and shoulder function?**

## DISCUSSION

First described by the Italian surgeon Iginio Tansini^1^ in 1896, the LD myocutaneous flap became popular in the late nineties as a consistently reliable option for postmastectomy autologous breast reconstruction. The large-caliber thoracodorsal artery and vein provide the LD flap with a long vascular pedicle that supplies both the muscle and overlying skin. This allows for the harvesting of a skin paddle that is versatile in both size and design to fill the skin defect from mastectomy. Once transferred, the underlying muscle not only adds volume to the breast, but also obviates dead space by mimicking the physiologic actions of the missing pectoralis major.^2^

A key caveat, however, is that the LD does not avail itself to a high-volume flap and is best suited for the reconstruction of small- to medium-sized breasts. To compensate for insufficient bulk and lack of projection to the inferior pole, the LD flap is often supplemented with tissue expanders or implants to increase soft-tissue coverage and improve aesthetic results. In addition, there is the high rate of postoperative seromas, occurring at anywhere from 20% to 79% of donor sites following traditional layered closure. However, the use of progressive tension sutures has been cited as a potential strategy to prevent prolonged drainage and seroma formation.^3^ Additional complications include hematoma, infection, fat necrosis, and flap loss.^4^^-^6

At present, the LD flap remains a steady fixture in the plastic surgery repertoire, even as the transverse rectus abdominis musculocutaneous (TRAM), and deep inferior epigastric perforator (DIEP) flaps have emerged as the gold standard in autologous breast reconstruction.^7^ The LD flap is not generally used for preliminary reconstruction given its lack of appropriate volume. It does, however, lend itself to being used as a salvage flap in instances of initial flap failure or expander/implant extrusion.^8^ Candidates for LD flap reconstruction include those for whom the TRAM or DIEP flaps are contraindicated, reveal a prior history of abdominoplasty, are active smokers, or present with medical comorbidities that preclude potentially lengthy microsurgical procedures. Patients in need of bilateral reconstruction may also choose not to use the TRAM or DIEP flaps because of their significant associated risks of donor site and flap complications and extended recovery times.^9^

Preoperatively, a physical examination should be conducted to assess the perfusion status of the LD. Many breast cancer patients will have undergone axillary node dissection, and a subsequent inability to adduct the arms against resistance is a sign that the LD was denervated in the process. Once the neurovascular health of the LD has been verified, reconstruction may proceed.

In patients with small bodies or small breast size, the LD flap can be used alone for a purely autologous tissue reconstruction. However, because of the flap's small volume and limited projection, expanders or implants are often used to reconstruct the contour of the inferior pole when greater bulk is required. The most commonly described technique is to place an implant into a partial subpectoral pocket beneath the transplanted muscle. The inferior lateral aspect of the implant is thereby covered by the autogenous soft tissue, promoting a natural ptosis that matches a contralateral ptotic breast.

Expander inflation can then be modulated to account for LD flap atrophy, subsidence of swelling, and the effects of radiotherapy. Complete coverage of the implant with the LD muscle allows for an arguably more aesthetic, naturally ptotic reconstructed breast while preserving the subpectoral space for any future revision or salvage operations. This protocol has been administered with low and stable long-term risks of reoperation, but with a high risk of dorsal seroma.^4^

A major concern in the postoperative course is donor site morbidity and the recovery of shoulder function after LD flap harvest. Much of the literature provides evidence that the LD muscle can be removed with minimal long-term impact on adduction, extension, and internal rotation of the humerus. A 2008 prospective study of 22 LD flap recipients further demonstrated that there was no significant loss of shoulder strength, function, or range of motion (ROM) as compared to preoperative values. A few patients experienced a loosening of the shoulder joint after mastectomy and reconstruction, which was attributed to intraoperative scar tissue release. A minimal loss of strength was reported in the first 6 months of recovery, with return to preoperative strength in the next 6 months. A majority of study subjects reported no long-term impact on the level of daily activity. For most, a full year was needed to regain baseline quality of life. Accordingly, an overall return to preoperative baseline is deemed likely for the majority of LD flap recipients, with most of the recovery expected to occur during the 6- to 12-month postoperative period. Furthermore, patient satisfaction with the cosmetic outcome has been consistently high. A 2001 review of women showed that 90% of the 121 study subjects had no complaints at the donor site; 84% were pleased with the results of the surgery; 95% denied feeling restricted in their occupation, private life, or during sports activities.^6^
